# In patients with primary Sjögren’s syndrome innate-like MAIT cells display upregulated IL-7R, IFN-γ, and IL-21 expression and have increased proportions of CCR9 and CXCR5-expressing cells

**DOI:** 10.3389/fimmu.2022.1017157

**Published:** 2022-11-24

**Authors:** Anneline C. Hinrichs, Aike A. Kruize, Helen L. Leavis, Joel A. G. van Roon

**Affiliations:** ^1^ Department of Rheumatology & Clinical Immunology, University Medical Center Utrecht, Utrecht University, Utrecht, Netherlands; ^2^ Center for Translational Immunology, University Medical Center Utrecht, Utrecht University, Utrecht, Netherlands

**Keywords:** Sjögren’s syndrome (pSS), MAIT cells, CCR9/CXCR5 T cells, IL-7Rα (CD127), IL-18Rα

## Abstract

**Introduction:**

Mucosal-associated invariant T (MAIT) cells might play a role in B cell hyperactivity and local inflammation in primary Sjögren’s syndrome (pSS), just like previously studied mucosa-associated CCR9+ and CXCR5+ T helper cells. Here, we investigated expression of CCR9, CXCR5, IL-18R and IL-7R on MAIT cells in pSS, and assessed the capacity of DMARDs to inhibit the activity of MAIT cells.

**Methods:**

Circulating CD161+ and IL-18Rα+ TCRVα7.2+ MAIT cells from pSS patients and healthy controls (HC) were assessed using flow cytometry, and expression of CCR9, CXCR5, and IL-7R on MAIT cells was studied. Production of IFN-γ and IL-21 by MAIT cells was measured upon IL-7 stimulation in the presence of leflunomide (LEF) and hydroxychloroquine (HCQ).

**Results:**

The numbers of CD161+ and IL-18Rα+ MAIT cells were decreased in pSS patients compared to HC. Relative increased percentages of CD4 MAIT cells in pSS patients caused significantly higher CD4/CD8 ratios in MAIT cells. The numbers of CCR9 and CXCR5-expressing MAIT cells were significantly higher in pSS patients. IL-7R expression was higher in CD8 MAIT cells as compared to all CD8 T cells, and changes in IL-7R expression correlated to several clinical parameters. The elevated production of IL-21 by MAIT cells was significantly inhibited by LEF/HCQ treatment.

**Conclusion:**

Circulating CD161+ and IL-18Rα+ MAIT cell numbers are decreased in pSS patients. Given their enriched CCR9/CXCR5 expression this may facilitate migration to inflamed salivary glands known to overexpress CCL25/CXCL13. Given the pivotal role of IL-7 and IL-21 in inflammation in pSS this indicates a potential role for MAIT cells in driving pSS immunopathology.

## Introduction

Primary Sjögren’s syndrome (pSS) is a systemic autoimmune disease clinically characterized by dryness of eyes and mouth, fatigue and myalgia (1). Hallmark features of pSS are B cell hyperactivity and lymphocytic infiltration of exocrine glands (2, 3). B cell hyperactivity is reflected by e.g. elevated serum IgG, the presence of autoantibodies in the circulation, elevated numbers of IgM/IgG plasma cells in the salivary glands, and an elevated risk of developing lymphoma (4, 5).

Several T cell subsets have been studied in pSS for their potential to drive B cell hyperactivity, and to organize ectopic lymphoid structures (ELS) (6–9).

T follicular helper (Tfh) cells are B cell activating T cells that express CXCR5, and that can reside in germinal centers (GCs) in lymph nodes (10). The number of Tfh cells is elevated in salivary glands and in peripheral blood of pSS patients, just like the levels of Tfh-associated cytokines such as CXCL13 (which CXCR5+ cells migrate towards), IL-21, and IL-4 (6, 7, 11–14). Tfh cell numbers correlate to autoantibody levels, and disease severity (14–18). Increased expression of ligand CXCL13 in the glands is associated with a higher number of lymphocytic foci and increased organization into ELS (6, 7, 9).

In the context of pSS also two Tfh-like cells subsets have been studied recently, T peripheral helper (Tph) cells, and CCR9+ Tfh-like cells, both lacking CXCR5. Tph cells are CXCR5-PD-1^hi^ memory T cells, produce high levels of IL-21 and CXCL13, like Tfh cells, and are found in elevated numbers in blood of pSS patients, and in the salivary glands of pSS patients with GCs in their gland tissue (18–20). CCR9+ Tfh-like cells are cells that are enriched in mucosa-associated tissues, and are potent B cell activating T cells that have elevated expression of ICOS, PD-1, CCL5 and IL-7R, and also produce high amounts of IFN-γ, and IL-21 (21–23). The number of CCR9+ Tfh-like cells is elevated in peripheral blood of pSS patients. In (mucosa-associated) salivary glands of pSS patients both CCR9+ T cell numbers, and levels of its ligand CCL25 are elevated (21–24). In secretomes of salivary gland tissue CCL25 levels were also found to be associated with SSA positivity, B cell hyperactivity (serum IgG levels), and levels of IL-21 and soluble IL-7R (25).

Other cells that are enriched in mucosal tissues and that are increasingly studied for their potential role in autoimmune diseases, including pSS, are mucosal-associated invariant T (MAIT) cells. MAIT cells are unconventional innate-like effector T cells that become activated by binding to MHC class I-like molecule MR1 (26). MAIT cells express a semi-invariant T cell receptor (TCR) TCRVα7.2-Jα12/20/33 with a limited amount of options of Vβ chains (27–30). MAIT cells are abundant in e.g. peripheral blood, liver, gastro-intestinal tract, and mesenteric lymph nodes, and play an important role in mucosal immunity against infections, e.g. by rapidly producing cytokines, such as IFN-γ, IL-21, TNF-α, IL-17, perforin, and granzyme B (31–37). MAIT cells are activated both in TCR-dependent and TCR-independent ways, including stimulation by IL-7, IL-12, and IL-18 (37–40). In humans MAIT cells predominantly express CD8, or express neither CD8 nor CD4 ( ± 70-80% and ±15%, respectively), and just a few percent of MAIT cells express CD4 (28, 35, 41, 42). MAIT cells can be identified in blood and tissues based on the expression of CD3 and TCRVα7.2 in combination with either CD161 and/or IL-18Rα (32, 35, 43). Furthermore high levels of CCR6, CXCR6, IL-7Rα (CD127), ABCB1 and NKG2D were found (35). Also CCR9-expressing MAIT cells have been identified, and were found to be enriched in mucosal sites such as the colon, with similar CCR9 expression between colonic CD8 MAIT cells and non-MAIT CD8 T cells (44). Finally MAIT cells in a human *in vitro* model have been shown to stimulate B cell activity associated with production of IL-21, IL-10, and IL-6, and in a lupus-like mouse model MAIT-driven B cell activation and autoantibody production has been shown (36, 45–47).

In pSS patients the number of circulating CD161+ TCRVα7.2+ MAIT cells was decreased compared to controls, whereas in salivary gland tissue the number of (CD161+) TCRVα7.2+ MAIT cells was elevated as compared to non-Sjogren sicca patients and mild sialoadenitis patients (without anousspSS) (48, 49). pSS patients had significantly more circulating CD4, and naive CD8 CD161+ TCRVα7.2+ MAIT cells, which was not the case for CD161- TCRVα7.2+ T cells (48). MAIT cells from salivary glands of pSS patients produced increased levels of IL-17, an effect that was validated *in vitro* upon stimulation of MAIT cells with IL-7 (49).

Currently, the number of studies on MAIT cells in pSS is limited and the precise mechanisms by which MAIT cells could instigate pSS immunopathology are not known. In the present study in pSS patients and healthy controls we for the first time investigated CCR9 (in comparison with CXCR5) as a receptor that might facilitate migration of MAIT cells to mucosa-associated tissues such as salivary glands. In addition, given the potential role of IL-7 and IL-18 to drive MAIT cell responses in immunopathology of pSS we here assessed the presence of IL-18Rα+ MAIT cells, and IL-18 and IL-7 receptor expression on MAIT cells in pSS patients as compared to HCs (50–53). Also the capacity of IL-7 to induce IL-21 and IFN-γ by MAIT cells was studied, as well as the potential of disease-modifying antirheumatic drugs (DMARDs) leflunomide and hydroxychloroquine to inhibit this production.

## Methods

### Patients and controls

Peripheral blood mononuclear cells (PBMCs) were collected from n=12 primary Sjögren’s syndrome (pSS) patients and n=11 healthy controls (HC). Controls were age and sex matched to patients. All pSS patients were diagnosed by a rheumatologist or clinical immunologist and met the 2016 ACR-EULAR criteria (54). All participants were included in the University Medical Center Utrecht (UMC Utrecht) and gave written informed consent. The Medical Research Ethics Committee (METC) of the UMC Utrecht approved the study (reference number 13/697). Demographic and clinical data are shown in **Table 1**.

**Table 1:** Participants’ characteristics.

	HC (n = 11)	pSS (n = 12)
Female, n (%)	11 (100)	12 (100)
Age, years	55 (49-60)	59 (54-64)
Anti-Ro/SSA positive, n (%)		8 (67)
Anti-La/SSB positive, n (%)		4 (33)
ANA positive, n (%)		7 (58)
Lymphocytic focus score (foci/4mm^2^)		1.8 (1.1-2.4)
IgA positive plasma cells (%)		65 (40-81)
Schirmer (mm/5min)		0 (0-1)
Serum IgG (g/L)		13.6 (11.4-15.2)
ESSDAI score (0-123)		4 (2-5)
ESSPRI score (0-10)		6.3 (5.3-7.3)
Immunosuppressants use, n		3

Medians with interquartile range (Q1-Q3) are shown, unless specified otherwise. Used immunosuppressants are hydroxychloroquine (n = 2) and methotrexate (n = 1). HC, healthy controls; pSS, primary Sjögren’s syndrome; anti-Ro/SSA, anti-Ro/Sjögren’s syndrome related antigen A antibody; anti-La/SSB, anti-La/Sjögren’s syndrome related antigen B antibody; ANA, antinuclear antibody; ESSDAI, European League Against Rheumatism (EULAR) Sjögren’s syndrome disease activity score; ESSPRI, EULAR Sjögren’s syndrome patient reported index.

Fresh PBMCs were isolated from Lithium-heparinized blood using density gradient centrifugation on Ficoll-PaqueTM Plus (GE Healthcare Life Sciences). Collected PBMCs were frozen and stored in liquid nitrogen until further use.

### Cultures

From 5 individuals (n=3 HC and n=2 pSS patients) 0,5.10^6^ thawed PBMCs were cultured overnight with optimal IL-7 concentrations as previously demonstrated at biologically relevant (10ng/ml, Peprotech) (55, 56). Leflunomide (33μM, biologically active metabolite A77 1726, MedChemExpress, Monmouth Junctions, USA), hydroxychloroquine (10μM, Sigma-Aldrich), or the combination of both drugs were tested at clinically relevant concentrations inducing optimal inhibition of lymphocyte activation as previously demonstrated (57, 58). In our hands upon *in vitro* cultures, and following T cell activation, CCR9 was strongly reduced after 48 hours of culture. Hence to appreciate cytokine secretion by CCR9+ T cells we cultured for 24 hours. For T cell cytokine analyses all samples were restimulated with phorbol myristate acetate (PMA) and ionomycin for 4 hours, in the presence of Brefeldin A. Medium control was taken along. The pSS patients whose PBMCs were used for this culture were not prescribed immunosuppressive drugs.

### Flow cytometry

PBMCs from all donors were thawed and stained with fixable viability dye eF780 (eBioscience), after which the cells were stained with fluorochrome-conjugated antibodies against CD3, CD4, CD8, CD45RO, CD161, CCR9, CXCR5, TCRVα7.2, IL-7Rα (CD127), and IL-18Rα (CD218α). For intracellular staining after overnight stimulation cells were fixed/permeabilized using Fixation/Permeabilization Concentrate and Diluent (Cat #00-5123-43, #00-5223-56, eBioscience) according to manufacturer’s protocol. In this panel IL-7Rα and IL-18Rα were replaced with IL-21 and IFN-γ. Details of used antibodies can be found in **Supplementary Table 1**. All samples were acquired on a BD LSRFortessa (BD Biosciences) using BD FACSDiva software v.8.0.1 (BD Biosciences). For data analysis FlowJoTM Software v10.8 (BD Life Sciences) was used. MAIT cells were defined using either CD161+ or IL-18Rα+, combined with TCRVα7.2+ CD3 cells. Gating of MAIT cells (CD161+ and IL-18Rα+ in combination with TCRVα7.2+) within CD4/CD8 populations is shown in **Supplementary Figure 1**.

### Statistical analysis

Data were analyzed and visualized using IBM SPSS Statistics 26 and Graphpad Prism 8. Mann-Whitney U test (for unpaired analyses) and Wilcoxon non-parametrical paired test were used (for paired analyses). For correlations Spearman’s rho was used. Statistical significance was considered for differences at p<0.05.

## Results

### The number of CD161 and IL-18Rα MAIT cells is decreased in pSS patients, and MAIT cells have a significantly different CD4/CD8 ratio in pSS patients

**Figure 1:**
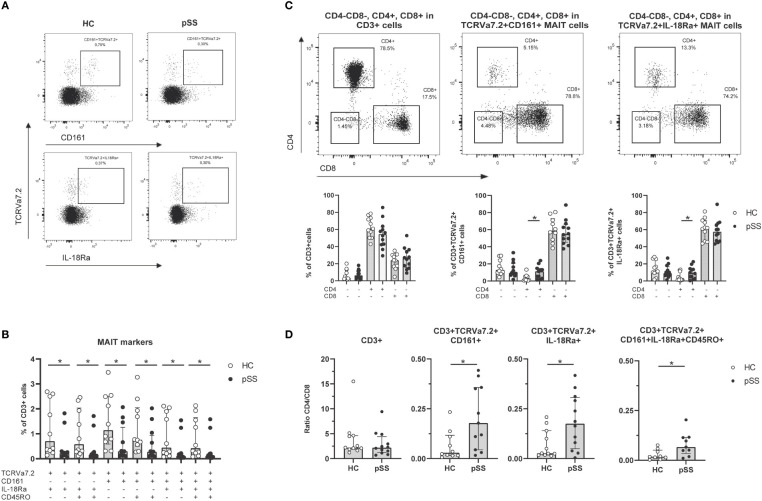
Reduced frequencies and shift in CD4/CD8 ratio of antigen-experienced CD161+/IL-18Rα+ MAIT cells in pSS patients. **(A)** Representative flow cytometry plots from a HC and a pSS patient of MAIT cell staining. Gates are shown of TCRVα7.2+CD161+ and TCRVα7.2+IL-18Rα+ within CD3+ cells. **(B)** MAIT cell subsets defined by TCRVα7.2 expression in combination with CD161, IL-18Rα, and CD45RO, are shown as percentages of the CD3+ T cell population in pSS patients and controls. **(C)** Representative dot plots of CD4/CD8 staining of all CD3+ cells, CD161+ and IL-18Rα+ TCRVα7.2+MAIT cells, with quantification below the representative dot plots. **(D)** CD4/CD8 ratio in CD3+ cells and MAIT cells defined by TCRVα7.2+CD161+, TCRVα7.2+IL-18Rα+, and combination of MAIT markers TCRVα7.2, CD161, IL-18Rα, and CD45RO in pSS patients and HC. Bar plots show all individuals and median (interquartile range). HC, healthy control; pSS, primary Sjögren’s syndrome. * indicates statistical significance of p < 0.05.

To define MAIT cells from live CD3 cells TCRVα7.2+ expression was analyzed together with either CD161+ or IL-18Rα+ (representative plots from HC and pSS patient in **Figure 1A**). The numbers of CD161+, IL-18Rα+, and co-expressing (memory) MAIT cells were all significantly decreased in pSS patients compared to HC (**Figure 1B**). Whereas in the total CD3 population CD4 T cells were the largest subset, in MAIT cells CD8 cells represented the largest subset, followed by CD4-CD8- and CD4 MAIT cells (**Figure 1C**). Increased numbers of CD4 MAIT cells were found in pSS patients as compared to controls (p=0.02 for CD161+, and p=0.049 for IL-18Rα+ MAIT cells, **Figure 1C**). This resulted in significantly skewed CD4/CD8 ratios for CD3 cells and MAIT cells from 2.2 (1.7-4.6) (median with interquartile range using pooled HC and pSS data) for CD3 cells to below 0.50 (median) in MAIT cell subsets **(** **Figure 1D**). In addition, in pSS patients a significantly higher CD4/CD8 MAIT cell ratio compared to HC was observed, in line with the increased CD4 MAIT cell percentages in pSS patients (**Figure 1D**).

### CCR9-expressing MAIT cells are mainly observed in CD4 MAIT cells and are rare in CD4-CD8- and CD8 MAIT cell populations

**Figure 2:**
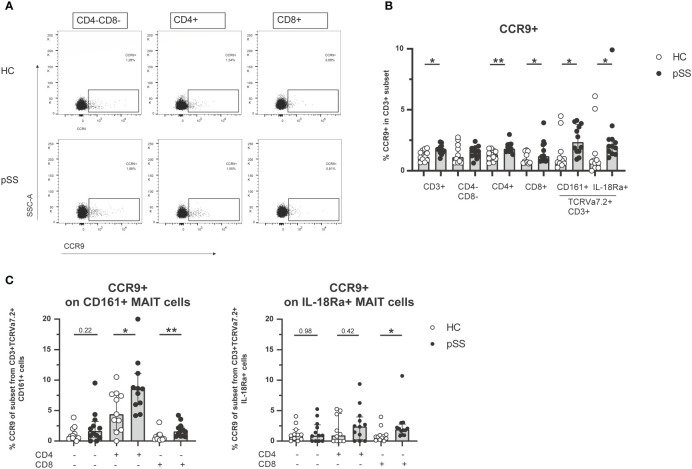
Increased frequencies of CCR9-expressing cells among circulating MAIT cells in pSS patients. **(A)** Representative flow cytometry plots of CCR9 expression on CD4-CD8-, CD4+, and CD8+ T cells from one pSS patient and one HC. **(B)** CCR9 expression is compared between pSS patients and healthy controls in different CD3+ cell subsets, including CD161 and IL18Ra-expressing MAIT cells. **(C)** In CD4-CD8-, CD4+, CD8+ cells from CD161+ and IL-18Rα+ MAIT cells CCR9 expression is quantified. Bar plots show median (interquartile range). HC, healthy control; pSS, primary Sjögren’s syndrome. *, ** indicates statistical significance of p < 0.05, p < 0.01, respectively.

Earlier work from our group and others has shown increased CCR9 expression on CD4 and CD8 T cells in pSS compared to HC (21–24). In this study we confirmed these findings (representative dot plots in **Figure 2A**). In fact, in most CD3 populations (CD3 total, CD4 T cells, CD8 T cells, CD161+ MAIT cells and IL-18Rα+ MAIT cells) the percentage of CCR9-expressing cells was elevated in pSS patients (**Figure 2B**). Within both CD161+ and IL-18Rα+ MAIT cell subsets CD4 cells expressed most CCR9. In addition, significantly increased CCR9 expression in pSS patients was found for CD161+ and IL-18Rα+ CD8 MAIT cells (**Figure 2C**). Overall the proportions of CCR9-expressing cells follow the same skewing in CD4-CD8-, CD4, and CD8 MAIT cells as in the respective total populations. Interestingly, for CXCR5 expression similar results were found. CXCR5 expression was elevated in pSS compared to HC in CD161+ and IL-18Rα+ MAIT cell subsets, with the highest expression of CXCR5 in CD4 MAIT cells (**Supplementary Figures 2, 3**).

### IL-7R expression is increased on CD8 MAIT cells, and is associated with inflammatory parameters. IL-21 production by MAIT cells is increased, and in the context of IL-7 activation reduced by LEF and HCQ

**Figure 3:**
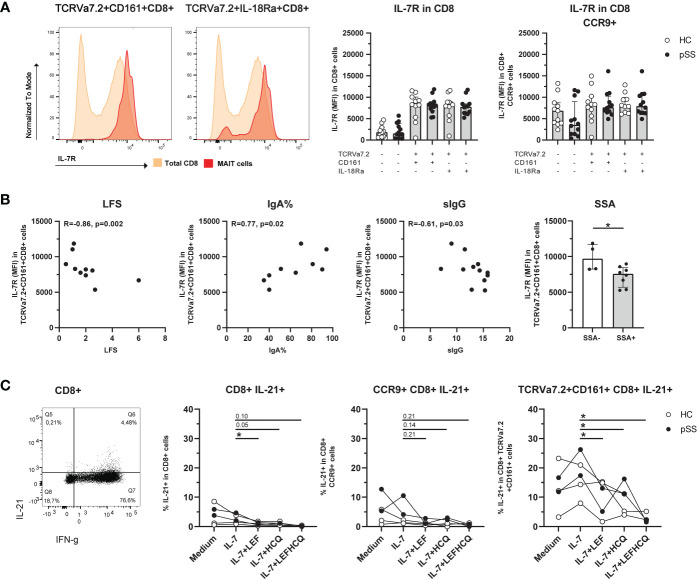
Expression of IL-7R is increased on CD8 MAIT cells, and this is associated with inflammatory parameters. IL-21 production by MAIT cells is increased and in IL-7-stimulated conditions reduced by leflunomide and hydroxychloroquine. **(A)** Representative histograms of IL-7R expression on CD8+ CD161+ and IL-18Rα+ TCRVα7.2+ MAIT cells compared to IL-7R expression in CD8 total population. Bar plots showing medians (interquartile range) of IL-7R on CD8+ (MAIT) cells and CCR9+ CD8+ (MAIT) cells in pSS patients and controls. **(B)** Correlation between IL-7R expression on CD8+ TCRVα7.2+CD161+ MAIT cells and local (LFS and percentage of IgA-producing plasma cells), and systemic (sIgG) clinical parameters. Bar graph shows comparison of IL-7R expression on CD8+ TCRVα7.2+CD161+ MAIT cells in pSS patients who are SSA autoantibody positive or negative. **(C)** Representative dot plot of IFN-γ/IL-21 expression in CD8+ cells. IL-21 expression of CD8+ cells, CCR9+ CD8+ cells, and CD8+ TCRVα7.2+CD161+ MAIT cells was measured upon overnight stimulation with IL-7, or IL-7 in combination with either leflunomide, hydroxychloroquine or a combination of both drugs. HC, healthy control; pSS, primary Sjögren’s syndrome; LFS, lymphocytic focus score; IgA%, percentage of IgA+ plasma cells in minor salivary gland biopsy (the percentage reduces when the percentage of IgM and IgG-producing plasma cells increases); sIgG, serum immunoglobulin G; SSA, anti-Sjögren’s syndrome related antigen A antibody; LEF, leflunomide; HCQ, hydroxychloroquine. * indicates statistical significance of p < 0.05.

CD8 MAIT cells showed a significantly increased IL-7R expression compared to the total CD8 population (**Figure 3A**). This increase was also seen in CD3, CD4-CD8-, and CD4 MAIT cells (**Supplementary Figure 4**). Of note, CCR9+ CD8 T cells showed a significant increase in IL-7R expression (p<0.001) compared to CCR9- CD8 T cells, which was not the case for IL-7R expression of CCR9+ CD8 MAIT cells compared to CCR9- CD8 MAIT cells, showing comparable high expression (**Figure 3A**). For CXCR5-expressing MAIT cells a similar pattern of IL-7R expression was seen as for CCR9+ cells (**Supplementary Figure 5**).

In pSS patients, a decreased expression of IL-7R on CD8 CD161+ MAIT cells significantly correlated to increased lymphocytic focus scores (LFS), decreased percentage of IgA+ plasma cells in minor salivary gland tissue (IgA%), and increased serum IgG levels (**Figure 3B**). Also, IL-7R expression on CD8 CD161+ MAIT cells was significantly decreased in pSS patients with anti-SSA-autoantibodies (**Figure 3B**).

Since DMARDs leflunomide (LEF) and hydroxychloroquine (HCQ) were recently shown to be efficacious *in vivo*, and *in vivo* block inflammatory activity (58), we tested the capacity of these drugs to target MAIT activity. For this purpose we cultured ex vivo IL-7-stimulated cells with LEF and HCQ, and evaluated IFN-γ and IL-21 production (Representative stainings **Supplementary Figure 6**). CD8 CD161+ MAIT cells produced more IL-21 compared to (CCR9+) non-MAIT CD8 T cells (median 12.2% versus 3.8% (p=0.009) and versus 5.4 (p=0.048) for CD8 and CCR9+ CD8 T cells respectively, **Figure 3C**). Unfortunately, it was not feasible to directly evaluate CCR9-expressing CD8 CD161+ MAIT cells due to limited CCR9 cell counts within this population. However, the results of CD8 T cells, CCR9+ CD8 T cells and CD8 CD161+ MAIT cells could be evaluated and showed a similar reduction in IL-21 expression in the presence of LEF and/or HCQ, which was statistically significant for conditions in MAIT cells (**Figure 3C**). Like IL-21, IFN-γ was also significantly higher in CD8 CD161+ MAIT cells as compared to CD8, and CD8 CCR9+ cells (mean 84.9% versus 55.0% (p=0.004), and 31.6% (p=0.003), respectively). However, in this short-term culture no significant effect on IFN-γ production was observed in response to LEF/HCQ treatment (data not shown).

## Discussion

In this study we demonstrated decreased circulating CD161+ as well as IL-18Rα+ MAIT cell numbers in pSS patients. In addition, we found that proportions of CCR9-expressing cells are elevated in MAIT cells of pSS patients. Interestingly, strongly increased CCR9 frequencies were observed in CD161-expressing, but not for IL-18Ra-expressing, MAIT cells. Furthermore, expression of IL-7R was found to be increased on CD8 MAIT cells as compared to total CD8 T cells. The expression of IL-7R on CD161+ MAIT cells correlated to the LFS, the percentage of IgA+ plasma cells in minor salivary gland tissue, and serum IgG levels, and was associated with the presence of anti-SSA antibodies. IL-21 production by CD8 MAIT cells stimulated with IL-7 was significantly reduced by LEF and HCQ treatments.

In line with earlier reports we found reduced CD161+ MAIT cells, which are at least partly caused by increased migration to inflammatory sites, since MAIT cells were found increased in the salivary glands of pSS patients as compared to controls (48, 49). In addition, we for the first time demonstrate that CD161/IL-18Rα co-expressing and single IL-18Rα+ MAIT cells are decreased in pSS patients compared to controls. Also, as anticipated CD161+ MAIT cells express high levels of the IL-18Rα as compared to non-MAIT cells, but this was not significant between pSS patients and HC (**Supplementary Figure 7**). This suggests that IL-18Rα+ MAIT cells/IL-18Rα expression may be mainly affected at the inflammatory sites. In pSS patients increased local IL-18 production by macrophages, dendritic cells, and epithelial cells has been demonstrated and was associated with increased immune activation (50, 51, 59). This indicates that increased numbers of IL-18Rα-expressing MAIT cells at the inflammatory sites in pSS could form another target for IL-18-mediated immune activation.

In accordance with earlier work, we found that in pSS patients the number of CD4 MAIT cells is elevated as compared to controls (48). Our data corroborate previous studies demonstrating that as compared to CD3 T cells MAIT cells have a strongly skewed CD4/CD8 T cell ratio with skewing towards CD8 T cells (28, 35, 41, 42). Here we demonstrate that for both CD161 and IL-18Rα-expressing MAIT cells in pSS patients this is even further skewed. As compared to non-MAIT CD8 cells CD8 MAIT cells produce high levels of IFN-γ and IL-21. Currently, it is unclear to what extent CD8 T cells and CD8 MAIT cells contribute to immune activation in pSS patients. Nonetheless, it was observed that CD8 T cells were found to associate with lymphocyte aggregates and Tfh numbers in salivary glands of pSS patients (24). Hence, we anticipate that increased CD8 MAIT cells may also significantly contribute to immune activation in the glandular lymphocytic foci given their effector profile. In addition, their innate-like properties, responding to specific environmental/infectious antigens suggests their capacity to respond in an early phase, perhaps (in certain cases) playing a crucial role in initiating immune responses.

Overexpressed CCL25 in salivary glands is associated with the presence of SSA autoantibodies and increases in lymphocyte foci, B cell hyperactivity, and salivary gland secretome IL-21 and sIL-7R levels (22). We here demonstrate that together with increased proportions of CCR9-expressing total CD4 and CD8 T cells, also proportions of CCR9-expressing CD161+ and IL-18Rα+ CD8 MAIT cells were significantly increased in pSS patients. Interestingly, the strongest increases in CCR9-expressing cells were found in CD4 CD161+ MAIT cells in pSS patients, which was not observed in IL-18Rα+ CD4 MAIT cells. The elevated levels of CCR9 expression in circulating (memory) MAIT cells of patients with pSS, a disease with inflammation in mucosa-associated tissues, fit to the observation that colonic MAIT cells with an activated memory phenotype express CCR9 and other chemokine receptors such as CCR6 (44).

In a similar pattern as CCR9-expressing MAIT cells, increased proportions of CXCR5-expressing MAIT cells were found. CXCR5 expression on CD161+ and IL-18Rα+ MAIT cells was significantly increased in pSS patients. CXCL13, the ligand for CXCR5, is overexpressed in salivary glands of pSS patients, and increased expression of CXCL13 is associated with increased B cell hyperactivity, and development of B cell lymphoma (6, 7, 60). Together this suggests that overexpressed CCL25 and CXCL13, facilitating the migration of CCR9 and CXCR5-expressing MAIT cells, in addition to classical Tfh cells and CCR9 Tfh-like cells, could contribute to immunopathology in pSS patients.

Significantly increased IL-7R expression was observed on MAIT cells, not significantly different between HC and pSS patients. Interestingly, reduction of IL-7R expression on CD8 MAIT cells was associated with local and systemic immune parameters, including lymphocytic focus scores and B cell hyperactivity. Since IL-7 causes IL-7R receptor downregulation this observation could reflect increased IL-7 levels in pSS patients (61, 62). Both systemically and in the inflamed glands of pSS patients increased IL-7 was associated with inflammatory parameters, and disease activity parameters (52, 53, 63). Alternatively, TCR-induced activation, which is another trigger of IL-7R downregulation, might be related to reduced IL-7R expression (64). Thus IL-7R downregulation might reflect activation of MAIT cells either *via* IL-7 stimulation or TCR cross-linking.

Immunohistochemical assessment of CCR9 and CXCR5 expression, as well as CD161 and IL-18Rα expression, on MAIT cells in the salivary gland of pSS patients is currently lacking. This might however be challenging due to low cell frequencies, but also due to potential downregulation of chemokine receptor CCR9. In our hands CCR9 expression was strongly reduced upon T cell activation after 48 hours of *in vitro* culture. Thus, considering immune activation and binding to its ligand CCL25 (which is elevated in pSS salivary gland tissue), assessment of CCR9+ T cells in inflamed salivary glands may be a challenge. The same uncertainty holds true for MAIT cell markers. For circulating cells it has been established that most MAIT cells will be identified using CD3 TCRVα7.2+CD161+ expression (28). Whether this is also the case for MAIT cells in salivary gland tissue of pSS patients still needs to be confirmed. Also, which subset of MAIT cells predominates locally (CD4, CD8 or CD4-CD8-) in pSS patients still is unknown. Enrichment of CD4-CD8- cells up to 40-50% of MAIT cells in mucosal tissues previously has been demonstrated and could match with the low frequencies seen in blood (65, 66). However, this still needs to be evaluated in pSS.

In this study we have demonstrated that MAIT cells in the circulation of pSS patients are antigen-experienced effector T cells that are characterized by high expression of IL-7R, IFN-γ, and IL-21. The increased proportion of CCR9 and CXCR5-expressing MAIT cells, and overexpression of the ligands CCL25 and CXCL13 to facilitate their migration into the inflamed tissues in these patients suggests that these cells with innate properties might contribute to the immunopathology in pSS. In this respect IL-21 a potent B cell activating factor could play a key role in B cell hyperactivity in pSS. Our data suggest that at least part of the activity of both CD8 and CD4 MAIT cells can be targeted by DMARD treatment such as leflunomide and hydroxychloroquine combination therapy.

## Data availability statement

The raw data supporting the conclusions of this article will be made available by the authors, without undue reservation.

## Ethics statement

The studies involving human participants were reviewed and approved by Medical Research Ethics Committee (METC) of the UMC Utrecht (reference number 13/697). The patients/participants provided their written informed consent to participate in this study.

## Author contributions

AH, AK, HL and JR were involved in conception and design of the study. AH, AK and HL were involved in data acquisition. AH, AK, HL and JR were involved in data analysis and interpretation. AH drafted the manuscript. All authors contributed to the article and approved the submitted version.

## Funding

AH is supported by ReumaNederland, grant number 17-2-403.

## Acknowledgments

We’d like to thank all participating patients and controls, and the Core Flow Facility of the Center for Translational Immunology from the UMC Utrecht.

## Conflict of interest

The authors declare that the research was conducted in the absence of any commercial or financial relationships that could be construed as a potential conflict of interest.

## Publisher’s note

All claims expressed in this article are solely those of the authors and do not necessarily represent those of their affiliated organizations, or those of the publisher, the editors and the reviewers. Any product that may be evaluated in this article, or claim that may be made by its manufacturer, is not guaranteed or endorsed by the publisher.
