# Supplementary material for: In patients with primary Sjögren’s syndrome innate-like MAIT cells display upregulated IL-7R, IFN-γ, and IL-21 expression and have increased proportions of CCR9 and CXCR5-expressing cells
Source: Front Immunol. 2022 Nov 24;13:1017157. doi: 10.3389/fimmu.2022.1017157 (PMC9729251; doi:10.3389/fimmu.2022.1017157)
Supplement: Supplementary file 1 [file DataSheet_1.docx]

Supplementary Material

## Supplementary Tables

**Supplementary Table 1. Antibodies used for flow cytometry.**

| Marker | Fluorochrome | Company | Clone |
| --- | --- | --- | --- |
| CD3 | AF700 | Sony Biotechnology | UCHT1 |
| CD4 | BV785 | Biolegend | RPA-T4 |
| CD8 | PE | Biolegend | RPA-T8 |
| CCR9 | APC | Biolegend | L053E8 |
| CXCR5 | PerCP-Cy5.5 | Biolegend | J252D4 |
| CD45RO | BV711 | BD Horizon | UCHL1 |
| CD127 (IL-7Rα) | PE | BD Phamingen | HIL-7R-M21 |
| CD161 | BV510 | BD Horizon | DX12 |
| TCRVα7.2 | PE-Cy7 | Biolegend | 3C10 |
| IL-18Rα (CD218α) | FITC | eBioscience | H44 |
| IL-21 | PE | BD Pharmingen | 3A3-N2.1 |
| IFN-γ | FITC | BD FastImmune | 25723.11 |
| Fixable Viability Dye | eFluor™ 780 | eBioscience | n.a. |

## Supplementary Figures

**
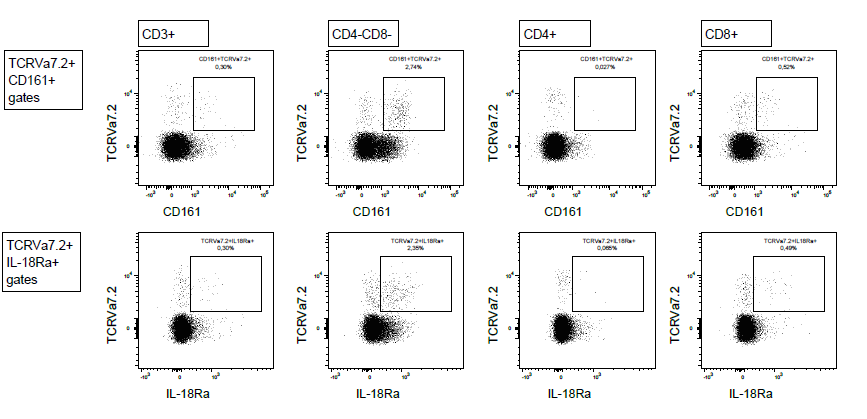
**

**Supplementary Figure 1.** **Gating strategy MAIT cells.** Representative flow cytometry plots and gating of CD161+ and IL-18Rα+ TCRVα7.2+ MAIT cells within CD3, CD4-CD8-, CD4+, and CD8+ T cells from a representative pSS donor.

**Supplementary figure 2. Increased frequencies of CXCR5-expressing cells among circulating MAIT cells in pSS patients.** (**A**) CXCR5 expression in TCRVα7.2+CD161+ MAIT cells, and CD4/CD8 TCRVα7.2+CD161+ MAIT subsets. (**B**) CXCR5 expression in TCRVα7.2+IL-18Rα+ MAIT cells, and CD4/CD8 TCRVα7.2+IL-18Rα+ MAIT subsets. *HC: healthy control; pSS: primary Sjögren’s syndrome.* indicates statistical significance of p<0.05.*

**
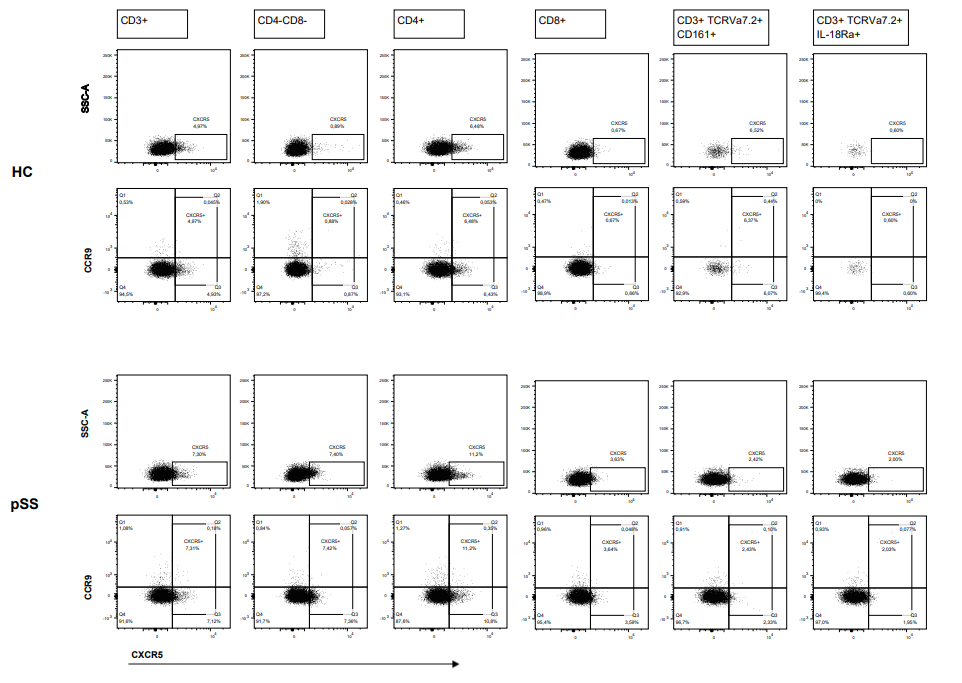
**

**Supplementary figure 3. Representative flow cytometry plots for CXCR5 staining of MAIT and non-MAIT T cells.** Representative dot plots of one HC and one pSS patient. Staining in CD3+, CD4-CD8-, CD4+, CD8+, TCRVα7.2+CD161+, and TCRVα7.2+IL-18Rα+ populations. *HC: healthy control; pSS: primary Sjögren’s syndrome.*

**
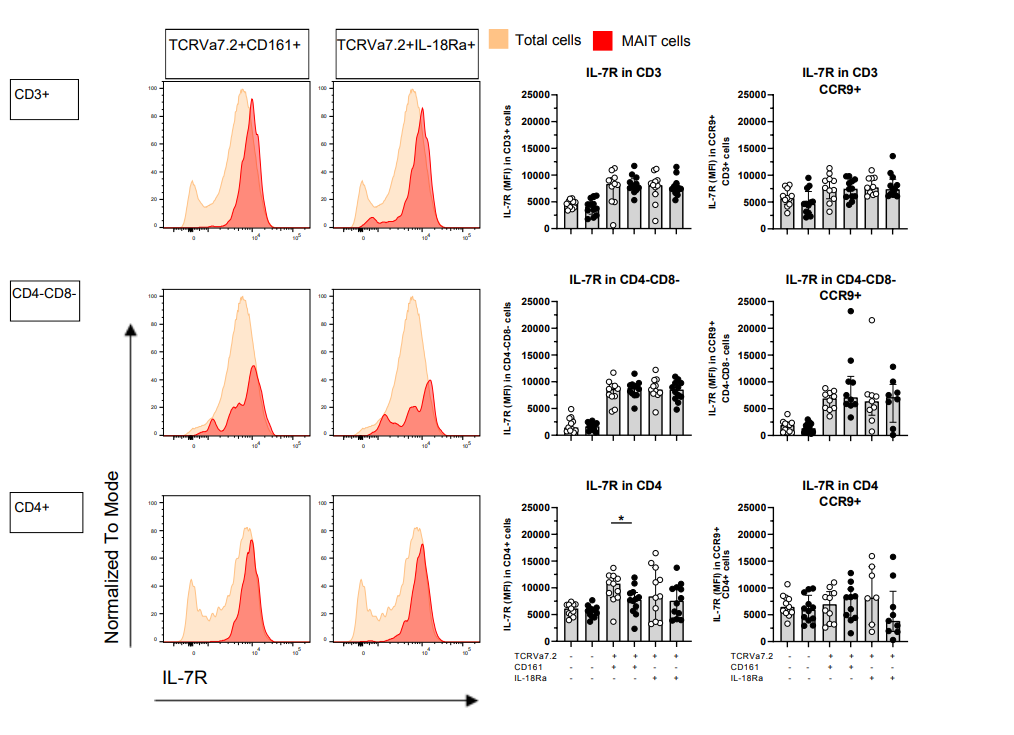
Supplementary figure 4. Expression of IL-7R is increased in CD3, CD4-CD8- and CD4MAIT cell subsets.** Representative histograms of IL-7R expression on CD3+, CD4-CD8-, and CD4+ CD161+ MAIT cells, and IL-18Rα+ MAIT cells compared to IL-7R expression on CD3, CD4-CD8-, and CD4+ total populations. Bar plots showing medians (interquartile range) of IL-7R on CD3, CD4-CD8-, and CD4 cells, and related CCR9+ (MAIT) cell populations in pSS patients and controls. *HC: healthy control; pSS: primary Sjögren’s syndrome.* indicates statistical significance of p<0.05.*

**
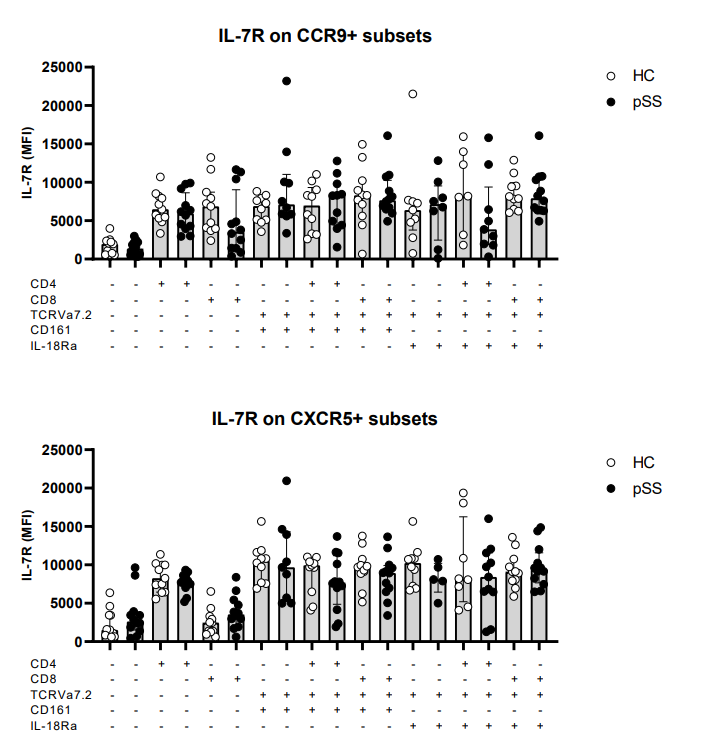
Supplementary figure 5. Expression of IL-7R is similar in CCR9+ and CXCR5+ MAIT cells.** Expression of IL-7R on CCR9-expressing cells in CD4/CD8-defined cell subsets, and in CD4/CD8-defined CD161+ and IL-18Rα+ MAIT cell subsets. Bar graphs show medians with interquartile ranges. *HC: healthy control; pSS: primary Sjögren’s syndrome.*


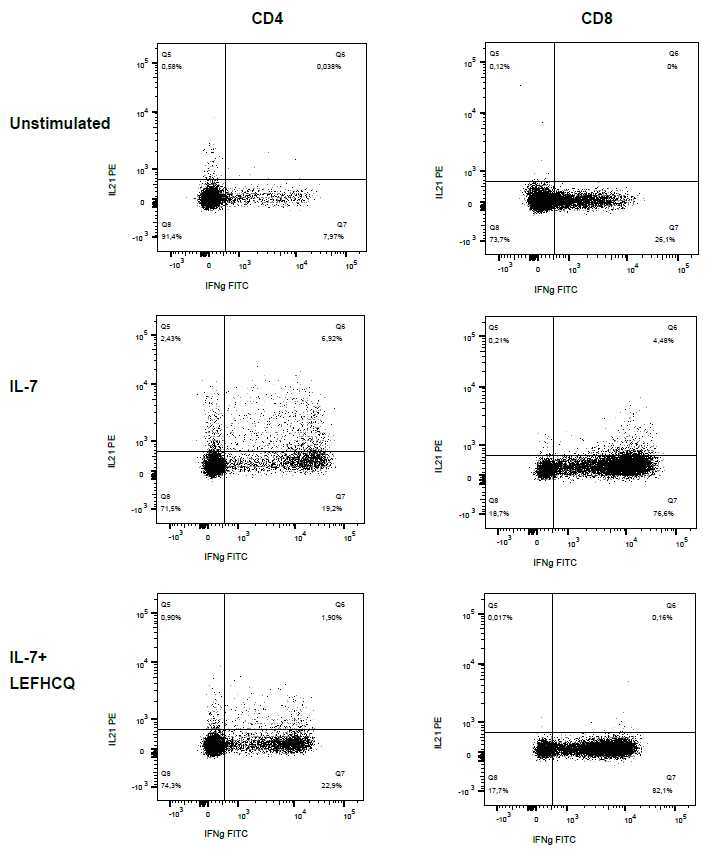


**Supplementary figure 6. Expression of IL-21 and IFN-γ in CD4 and CD8 cells under different culture conditions.** Dot plots of unstimulated cells, IL-7 stimulated cells and IL-7 stimulated cells in the presence of leflunomide and hydroxychloroquine are shown for CD4 and CD8 T cells.

*
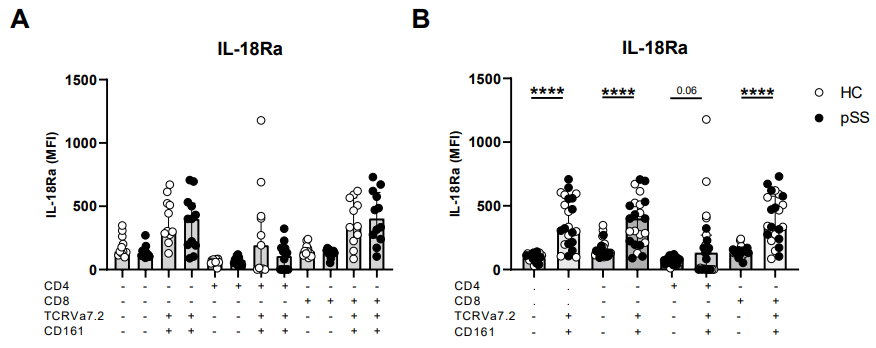
***Supplementary figure 7. Expression of IL-18Rα is similar between pSS patients and controls, and elevated in MAIT cells compared to non-MAIT cells.** (**A**) Expression of IL-18Rα on CD4-CD8-, CD4+, and CD8+ non-MAIT and CD161+ MAIT cells in pSS patients and HC. (**B**) Expression of IL-18Rα on CD3+, CD4-CD8-, CD4+, and CD8+ non-MAIT and CD161+MAIT cells, data from pSS patients and HC pooled.

Bar graphs show medians with interquartile ranges. *HC: healthy control; pSS: primary Sjögren’s syndrome.**** indicates statistical significance of p<0.0001.*
